# Nutrient-Driven Antioxidant Interventions for Prevention of Age-Related and Diabetic Cataracts

**DOI:** 10.3390/nu17111885

**Published:** 2025-05-30

**Authors:** Rosa Giglio, Serena Milan, Leandro Inferrera, Daniele Tognetto, Fabiana D’Esposito, Federico Visalli, Caterina Gagliano, Marco Zeppieri

**Affiliations:** 1 Department of Medicine, Surgery and Health Sciences, University of Trieste, 34127 Trieste, Italy; giglio.rosam@gmail.com (R.G.);; 2 Imperial College Ophthalmic Research Group (ICORG) Unit, Imperial College, 153-173 Marylebone Rd, London NW1 5QH, UK; 3 Department of Neurosciences, Reproductive Sciences and Dentistry, University of Naples Federico II, 80131 Napoli, Italy; 4 Department of Ophthalmology, University of Catania, 95123 Catania, Italy; 5 Department of Medicine and Surgery, University of Enna “Kore”, Piazza dell’Università, 94100 Enna, Italy; 6 Mediterranean Foundation “G.B. Morgagni”, 95100 Catania, Italy; 7 Department of Ophthalmology, University Hospital of Udine, 33100 Udine, Italy

**Keywords:** cataract prevention, oxidative stress, antioxidants, lens transparency

## Abstract

Cataract formation remains a significant cause of global visual impairment. Increasing attention has been directed toward antioxidant-based interventions as potential non-surgical strategies to delay or prevent cataractogenesis, particularly in the age-related and diabetic contexts. This review summarizes recent preclinical evidence on nutritional antioxidants for the prevention of age-related and diabetic cataracts. Agents such as trimetazidine, Moringa oleifera stem extract, ginsenoside Rg1, lanosterol nanoparticles, β-casomorphin-7, and cerium oxide-based nanotherapies have been shown to mitigate oxidative damage, modulate redox signaling pathways, and preserve lens clarity. Advances in drug delivery, including topical formulations, nanoparticle carriers, and intravitreal injections, have been proposed to overcome the anatomical and pharmacokinetic barriers associated with the avascular lens. The new data support ongoing translational research to maximize the clinical use of antioxidants and highlight their therapeutic potential in the prevention of age-related and diabetic cataracts.

## 1. Introduction

Cataracts remains one of the leading causes of visual impairment across the globe, with its prevalence steadily increasing alongside the aging population [1]. As the population ages and the prevalence of chronic conditions such as diabetes rises, the socio-economic and public health burdens associated with cataracts are increasing. Therefore, there is a growing interest in exploring non-surgical strategies to delay cataract onset and progression [2]. Age-related cataract remains the most prevalent form worldwide, but diabetic cataracts (DCs) are becoming increasingly common as well. The transparency of the natural lens, an avascular tissue, depends on a delicate balance of antioxidants. Oxidative stress is widely recognized as a key contributor to the pathogenesis of cataracts, primarily through its role in damaging lens proteins and lipids [3]. Both types of cataracts, age-related and DCs, share oxidative stress as a crucial pathogenic mechanism, yet they arise through partially distinct biological pathways. In DCs, chronic hyperglycemia introduces additional factors, such as the activation of the polyol pathway, advanced glycation end products (AGEs), and impaired antioxidant defense systems. Given the frequent co-occurrence of aging and diabetes, examining both age-related and DCs might offer a more comprehensive framework for evaluating antioxidant interventions [4].

Diet adjustments could be a cost-effective and non-invasive way to prevent lens opacification and cataract surgery, and save the associated costs. Dietary antioxidants (i.e., vitamins A, C, and E) and carotenoids (i.e., lutein and zeaxanthin) can neutralize reactive oxygen species (ROS) that lead to protein aggregation and, as a consequence, lens opacification. While surgical treatment remains the gold standard, identifying and clinically implementing nutritional strategies may have significant public health implications. In preclinical studies, oxidative stress has been shown to cause aggregation of lens proteins, specifically crystallins, which subsequently causes light scattering [2]. Cataractogenic inhibitors such as antioxidants have been shown to maintain lens transparency in vitro. Animal models, including streptozotocin-induced diabetic rats and UV-exposed mice, showed similar findings, suggesting that antioxidant supplementation may prevent the onset of cataracts and optimize antioxidant enzyme activity in the lens. Preclinical studies provided evidence for the biological function of antioxidants to prevent cataracts; however, this concept does not easily translate into clinical settings. Several studies have examined the association between antioxidant intake and cataract onset, providing valuable information on the possible protective role of dietary vitamins and carotenoids [3].

Antioxidant intake is inversely associated with cataracts in observational studies, but interventional trials are often conflicting. This difference might be due to differences in the study population, baseline nutritional status, supplementation dosage and formulation, complexity of nutrient absorption, and metabolism within the lens. Jiang et al. reported the association between dietary vitamin and carotenoid intake and incidence of age-related cataracts and proposed that a higher intake of vitamins A, C, and some carotenoids was inversely associated with the risk of cataracts in cohort studies, while randomized controlled trials (RCTs) showed more ambiguous findings [5]. A study of the nutritional implications of cataract development and progression was presented by Sella and Afshari in 2018 as well, recognizing both the encouraging observational data, but also the challenges faced when carrying out clinical intervention trials [2]. While antioxidants such as vitamins A, C, and E, and carotenoids such as lutein and zeaxanthin show promise, translation of their effects to reproducible clinical benefits has been compromised by heterogeneity of study design and variability of patient compliance, they noted. Braakhuis et al. (2019) extensively reviewed nutritional approaches to cataract prevention with a focus on the complex mechanisms involved in lens protection by antioxidants and, therefore, promoting their topical delivery via overcoming the anatomical and metabolic challenges conferred by the lens architecture [6,7].

The present work seeks to provide an updated overview of the literature, summarizing research that has not been addressed in prior reviews. We aim to identify new insights that have arisen regarding mechanisms through which lens transparency might be modulated. The current literature provides an evidence base for antioxidant interventions in cataract prevention (both age-related and diabetic) to identify gaps in the current research space and to suggest possible directions for the next set of research.

## 2. Materials and Methods

We performed a systematic search for all available articles exploring the role of antioxidants in cataractogenesis and cataract prevention. A literature search of all original articles published was performed in parallel by two authors (S.M. and L.I.) using the PubMed database. The following terms were employed (“Cataract/prevention and control”[MeSH Terms] OR “Cataract/etiology”[MeSH Terms]) AND (“Antioxidants/therapeutic use”[MeSH Terms] OR “Oxidative Stress/drug effects”[MeSH Terms] OR “Vitamins/pharmacology”[MeSH Terms] OR “Dietary Supplements/therapeutic use”[MeSH Terms] OR “Phytochemicals/therapeutic use”[MeSH Terms]) AND (“Free Radicals/metabolism”[MeSH Terms] OR “Risk Factors”[MeSH Terms] OR “Aging/physiology”[MeSH Terms] OR “Lifestyle”[MeSH Terms]) NOT “Surgical Procedures, Operative”[MeSH Terms].

After the preparation of the list of all electronic data captured, two reviewers (S.M. and L.I.) examined the titles and abstracts independently and selected relevant articles identified by the initial search using Rayyan QCRI online software. We decided to include articles published from July 2018 up to 7 April 2025. The timeframe of the literature search was defined in consideration of existing systematic reviews and meta-analyses, which had already provided a comprehensive synthesis of the literature up to that period. The full texts of the relevant articles were analyzed, and the bibliography of eligible articles was assessed to identify any studies not obtained through an electronic search.

Only original studies, both in vitro and in vivo, were included in the current review. Exclusion criteria were review studies, pilot studies, case reports, letters, photo essays, studies written in languages other than English, and causes of cataracts other than senility and diabetes. The same reviewers registered and selected the captured studies according to the inclusion and exclusion criteria by examining the full text of the articles. Any disagreement was assessed by consensus, and a third reviewer (R.G.) was consulted when necessary. The level and quality of evidence of the selected studies were evaluated based on the Oxford Centre for Evidence-Based Medicine (OCEM) (Oxford, UK) 2011 guidelines and the Grading of Recommendations Assessment, Development and Evaluation (GRADE) system, respectively [8,9]. The relative efficacy has been defined as Very High: Multifaceted mechanism, robust outcomes across multiple models; High: Consistent improvement in lens transparency and redox homeostasis; Moderate–High: Demonstrated efficacy in key parameters but limited in scope or model; and Moderate: Effective in early-stage cataract or partial restoration.

## 3. Results

From 90 articles extracted from the initial research, 11 abstracts were identified for screening, and 9 of these met the inclusion/exclusion criteria for full-text review. Studies are thematically organized in the following sections to emphasize trends and discrepancies in findings, accompanied by interpretive commentary that seeks to identify research gaps and future prospects. The results are reported in Table A1 (Appendix A).

### 3.1. Age-Related Cataractogenesis

#### 3.1.1. Trimetazidine

In vivo and in vitro studies have been performed to analyze the protective efficacy of trimetazidine (TMZ) in sodium selenite-induced cataractogenesis, a well-accepted model of oxidative stress-induced lens opacification [10]. TMZ, a metabolic modulator with established antioxidant properties, was found to delay or prevent cataract formation via modulation of redox signaling and epigenetic regulation. In vivo, sodium selenite was administered subcutaneously into suckling Sprague-Dawley rats, leading to the development of lens opacities. As a control group, a parallel treated group was also treated with intraperitoneal TMZ before selenite treatment. Cataract formation was evaluated by slit-lamp biomicroscopy and scored on a 6-point scale. TMZ was linked to a drastic decrease in lens opacification: treated rats showed, in comparison to naïve selenite treated ones, significantly milder cataract scores (Grades 1–3 versus Grades 4–6).

In vitro, human lens epithelial B3 (HLEB3) cells were treated with selenite and TMZ, and cellular assays validated that TMZ increased cell viability, decreased ROS generation, and mitigated apoptosis. The test demonstrated that selenite perturbed the Keap1/Nrf2 antioxidant signaling cascade, resulting in an overexpression of Keap1 and suppressed Nrf2-entering, a decrease in the expression of downstream antioxidant genes (superoxide dismutase -SOD-, glutathione peroxidase -GPx-, and catalase—CAT), and an upregulation of the markers of oxidative stress (malondialdehyde -MDA-, nitric oxide -NO-, and hydrogen peroxide- H_2_O_2_). Moreover, bisulfite genomic DNA sequencing analysis indicated that selenite treatment was associated with demethylation of CpG sites in Keap1 promoter, an epigenetic modification that caused Keap1 overexpression and disruption of the antioxidant system. It confirmed that TMZ treatment inhibited this demethylation, a possible epigenetic protective mechanism. Western blotting also indicated that TMZ restored the Bcl-2/Bax ratio and inhibited lens epithelial cell apoptosis [10].

#### 3.1.2. Moringa Oleifera Stem Extract

The protective potential of Moringa oleifera stem extract (MOSE) against H_2_O_2_-induced cataractogenesis has been shown using an ex vivo mouse lens organ culture system [11]. While prior studies had investigated the antioxidant properties of other parts of the Moringa plant, this study was the first to evaluate the stem extract, an agricultural byproduct, for its efficacy in delaying or preventing lens opacity through antioxidant mechanisms. Mouse lenses were pre-treated with MOSE (0.5 and 1.0 mg/mL added to culture medium) for 24 h, followed by exposure to 1 mM H_2_O_2_ to induce oxidative stress, and then incubated again for 48 h in standard medium. Lens transparency was assessed microscopically, and biochemical assays were conducted to measure ROS, reduced glutathione (GSH) levels and the activities of antioxidant enzymes (SOD and CAT). Additionally, the expression of peroxisome proliferator-activated receptor alpha (PPARα) was evaluated via western blotting, given its known regulatory role in redox homeostasis.

The results demonstrated that MOSE significantly reduced lens opacity and ROS accumulation, and the 1 mg/mL dose proved more effective than 0.5 mg/mL. MOSE also augmented GSH content and restored the enzymatic activities of SOD and CAT. Western blot analysis confirmed that MOSE upregulated the protein expression of SOD, CAT, and PPARα following oxidative insult. MOSE outperformed luteolin, a reference flavonoid compound, in enhancing endogenous antioxidant defenses despite showing lower direct free radical scavenging capacity in 2,2-Diphenyl-1-1-picrylhydrazyl (DPPH) assays.

#### 3.1.3. L-Cysteine

A new, low-cost method for monitoring cataractogenesis in cultured bovine lenses with light transmittance measurements and biochemical markers has been reported in the literature [12]. With traditional imaging techniques being complicated and costly, the authors hypothesized that a plate reader-based optical transmission assay at 420 nm together with the measurement of GSH and SOD activity could serve as a reliable method to evaluate lens opacity progression and treatment efficacy in vitro. In their experimental model, freshly isolated bovine lenses were cultured in Dulbecco’s Modified Eagle’s medium (DMEM) with or without H_2_O_2_, and exposed to either L-cysteine or ascorbic acid (AA) as positive controls. The lenses were incubated for up to 120 h, and light transmittance across 230–710 nm was measured at various intervals using a spectrophotometric plate reader.

The degree of opacity was then correlated with antioxidant enzyme activity, measured through ELISA-based assays. The results showed a time-dependent decrease in lens transparency in DMEM-cultured lenses, with the most evident drop occurring at 420 nm. Exposure to H_2_O_2_ accelerated lens opacification in a concentration-dependent manner, reducing transmittance by up to 87.7% at 100 mM. L-cysteine significantly reversed this trend, especially at concentrations of 10^−6^–10^−4^ M, showing better efficacy than AA in preserving lens clarity over 120 h. Biochemical data further supported the optical findings: both total GSH content and total SOD activity were substantially reduced after prolonged DMEM or H_2_O_2_ exposure. L-cysteine treatment significantly mitigated these declines, increasing GSH by up to 76.6% and restoring SOD activity by over 160% in oxidatively stressed lenses. These effects suggested that L-cysteine might counteract oxidative stress both by restoring sulfhydryl pools and by directly stabilizing antioxidant enzymes.

#### 3.1.4. Lanosterol Nanoparticles

A study in the literature assesses the therapeutic effect of lanosterol nanoparticles (LAN-NPs) administered via intravitreal injection in Shumiya Cataract Rats (SCRs), a hereditary model of lens degeneration [13]. Earlier findings had already proposed that lanosterol might prevent or reverse crystallin protein aggregation and restore lens transparency in animal models, though clinical relevance remained controversial. The authors hypothesized that a nanoparticulate formulation would improve lanosterol solubility, intraocular bioavailability, and therapeutic efficacy in early-stage cataracts. SCRs were classified into two subtypes: SCR-N, which showed mild age-related lens structural breakdown but no opacity, and SCR-C, which produced considerable opacification and posterior nuclear displacement after 12 weeks.

The bead-milling procedure was refined to create LAN-NPs with particle sizes ranging from 50 to 400 nm. The 0.5% LAN-NPs remained stable for two weeks and consisted predominantly of solid-phase lanosterol. The enhanced formulation exhibited no cytotoxicity in human lens epithelial cells (HLECs) and produced no apparent irritation, inflammation, or cloudiness in vivo. Repeated intravitreal injection of LAN-NPs (every 2 days for 6 weeks) resulted in sustained lanosterol levels in the lens for up to 48 h. In SCR-N rats, LAN-NPs reversed early lens structure collapse and maintained lens clarity. In contrast, in SCR-C rats, LAN-NPs delayed but did not halt or reverse lens opacification or the severe posterior movement of the nucleus. Histological analysis confirmed that the serious architectural damage in SCR-C lenses was not repaired by treatment. However, LAN-NPs attenuated key biochemical markers of cataract progression, including calcium accumulation, nitric oxide production, and lipid peroxidation (LPO). The study also addressed an ongoing debate in the literature: whether lanosterol directly dissolves crystallin aggregates or simply preserves lens homeostasis at early stages.

#### 3.1.5. Ginsenoside Rg1

The antioxidant and cytoprotective potential of Ginsenoside Rg1 (Rg1) in preventing H_2_O_2_-induced lens opacity has been studied, using an ex vivo rat lens organ culture model [14]. The study was based on the premise that oxidative stress represents a key trigger in cataractogenesis, particularly through LPO and diminution of endogenous antioxidants. Given Rg1’s known neuroprotective and anti-apoptotic properties, the authors aimed to explore its possible application in ophthalmic redox biology. In the experimental design, Sprague-Dawley rat lenses were cultured in Medium 199 and exposed to 0.2 mM H_2_O_2_, either alone or in combination with varying concentrations of Rg1 (ranging from 0.1 to 1.0 mM). Lenses were evaluated across multiple endpoints, including opacity grading, histological analysis, protein solubility, antioxidant enzyme activity, and GSH/MDA quantification.

The most effective concentration of Rg1 was found to be 0.6 mM, which was selected for further experimentation. Results demonstrated that lenses exposed to H_2_O_2_ alone exhibited severe opacity (Grades 3–4), histological disintegration of lens epithelial cells (LECs), and structural fiber degeneration. Rg1 treatment markedly reduced lens opacity, preserved lens architecture, and minimized pathological changes. Rg1 treatment also enhanced cell viability in HLECs exposed to oxidative stress, particularly at 0.4–0.6 mM concentrations. Moreover, Rg1-treated lenses showed significant increases in water-soluble protein (WSP) content, SOD activity, and reduced GSH levels, alongside a significant decrease in MDA and oxidized GSH, which are key markers of LPO and oxidative damage [14].

#### 3.1.6. *N*-Acetylcysteine

Ishida et al. described a multi-model study that evaluated the effect of *N*-acetylcysteine (NAC) and *N*-acetylcysteine amide (NACA) against age-associated cataractogenesis via oxidative stress and redox signaling pathways [15]. Acknowledging the prominent role of oxidative damage in lens opacification, particularly in elderly cohorts, the authors investigated NACA and NAC in vitro (HLECs and MLECs) and ex vivo (rat lens organ culture), as well as in vivo (aged mouse model), stressing translational applicability. The above topical formulation (2 mM) was examined on a 28-day timeframe.

In vitro NACA-treated cells exposed to oxidative stress (100 µM H_2_O_2_ or tBHP) significantly reduced ROS levels and enhanced cell survival rates. Additionally, NACA suppressed the overexpression of catalase and PRDX6 mRNA, suggesting decreased transcriptional activation due to ROS and therefore a dampened oxidative insult, as opposed to compensatory upregulation. Using an ex vivo rat lens model, we show that NACA pre-treatment prevents opacification in lenses exposed to H_2_O_2_ and preserves lens clarity. This was confirmed with significant improvements by image analysis and opacity quantification. Further validation in an in vivo model using 62-week-old C57BL/6 mice showed that both NAC and NACA suppressed natural age-related lens opacity; however, the molecular effect of NACA was dominant in vivo, specifically a more extensive suppression of the NFAT-regulated expression of thioredoxin-interacting protein (TXNIP) without altering the mRNA content of both PRDX6 and catalase. Histological assessment revealed intact cortical fiber structure in treated lenses.

### 3.2. Diabetic Cataractogenesis

#### 3.2.1. β-Casomorphin-7

The protective role of β-casomorphin-7 (β-CM-7), a milk-derived opioid peptide, has been reported on oxidative stress-induced damage in HLECs subjected to a high-glucose environment [16]. Since DCs are a rapid-onset and frequent complication of diabetes mellitus, the authors attempted to define whether β-CM-7 could exert antioxidant protection through the modulation of transcription factors such as FoxO1 and Sp1, known regulators of oxidative stress responses. This exploratory study was performed in vitro, in which the SRA01/04 HLEC line was cultured under hyperglycemic conditions (40 mM glucose during 48 h), before and after pretreatment with various concentrations of β-CM-7 (10^−5^ to 10^−8^ mol/L).

Authors assessed cell viability via 3-(4, 5-dimethyl-2-thiazolyl)-2, 5-diphenyl-2-H-tetrazolimol/L bromide (MTT) assay, the oxidative stress via flow cytometry, and ELISA-based biochemical assays, western blotting, and immunofluorescence were used for ROS, MDA, SOD, GSH-px and the expression/localization of Foxo1 and Sp1. High-glucose exposure resulted in lower HLEC viability, detectable intracellular ROS and MDA, and decreased antioxidant enzyme levels (SOD and GSH-px). At the same time, Foxo1 and Sp1 were downregulated, and the localization of Foxo1 was also diverted from the nucleus. β-CM-7 pretreatment reversed these effects significantly, especially at 10^−5^ mol/L: levels of ROS and MDA decreased, and SOD activity and GSH-px expression increased. Furthermore, Foxo1 was rescued and transfused into the nucleus, in addition to Sp1 expression in the nucleus being boosted.

#### 3.2.2. Rapamycin

The role of autophagy and oxidative stress in DC formation and their relationship has been assessed in studies by using mouse LECs exposed to high-glucose (HG) environments [17]. The studies aimed to investigate how chronic hyperglycemia influenced autophagic flux and redox homeostasis, both in vitro and in vivo. The experiments were conducted using streptozotocin-induced diabetic mice and mouse lens capsular bag cultures under high-glucose (HG) conditions. During a timeframe of 4 months in vivo and up to 72 h in vitro, studies of lens tissues as well as LECs were conducted for electron transmission microscopy, qRT-PCR, immunohistochemistry, western blotting, and mitochondrial ROS detection.

The authors performed treatment with rapamycin (an autophagy inducer) and chloroquine (autophagy inhibitor) to explore the causal relationships between autophagy activation and oxidative stress responses. The findings showed in LECs from a diabetic context a responsive autophagic response that was dysregulated. The autophagy marker LC3B increased early but plateaued out at later time points, whereas the substrate p62, which would be degraded during rapid autophagy, decreased and then later accumulated, reflecting a blockage of autophagy flux over time. Electromicroscopy confirmed the presence of larger autophagosomes in diabetic tissues, some containing damaged mitochondria. A biphasic oxidative stress response was detected.

Both in vivo and in vitro, mRNA levels of antioxidant enzymes SOD2 and CAT were upregulated during short-term HG exposure, then significantly reduced after prolonged exposure. Mitochondrial ROS production increased significantly by 72 h under HG conditions, indicating cumulative redox damage in the diabetic milieu. However, co-treatment with rapamycin markedly increased autophagic activity, whereas the levels of ROS significantly decreased, indicating that promoting autophagic activity may alleviate oxidative injury. Conversely, chloroquine treatment exacerbated oxidative damage and mitochondrial stress, strengthening the idea of autophagy as a protective process.

#### 3.2.3. Cerium Oxide Nanoparticles and Resveratrol

A nanomedicine approach to DC prevention using cerium oxide nanoparticles (CeO_2_ NPs) co-loaded with resveratrol (RSV) and exosome-derived microRNAs (miRNAs) has been examined in studies [18]. The composite system was designed to target the three deregulatory driving pathological features in DCs: oxidant stress, glycation stage of proteins, and disruption of gene members in regulatory networks. To overcome the limitations of classical single-target therapeutics, the authors engineered a multi-targeted nanosystem featuring antioxidant, antiglycative, and epigenetic components. In vitro assays performed on HLECs under HG conditions indicated the successful reduction in ROS, LPO (MDA and conjugated diene levels), and apoptosis due to miR-NA-RSV-CeO_2_ NPs.

The multifunctional nanoparticles were more effective than CeO_2_ NPs or RSV alone in maintaining cellular redox balance and viability. This response was driven primarily by a cerium oxide core that dynamically cycled between Ce^3+^ and Ce^4+^ states, thus disclosing self-regenerative antioxidant capacity. Besides measuring oxidative stress, production of advanced glycation end products, a major cause for lens opacification in diabetic conditions, was also investigated. The nanoparticles effectively inhibited the formation of advanced glycation end-products (AGEs) in various protein-glycation systems, including bovine serum albumin (BSA)-glucose, BSA-methylglyoxal, and hemoglobin-low-density lipoprotein (LDL) systems, with half-inhibitory concentration (IC_50_) values ranging from 123 to 209 µg/mL. These properties were mainly ascribed to RSV, which was better stabilized and delivered within the matrix of the nanoparticle. Importantly, it added a third dimension of therapeutic activity by embedding the exosome-derived miRNAs. The authors included a gene expression analysis showing that miRNA-RSV-CeO_2_ NPs upregulated CRYAA mRNA expression in HLECs, promoting the maintenance of α-crystallin (a molecular chaperone critical for lens transparency and protection against protein aggregation).

The miRNA component showed modulation of gene networks and pathways related to oxidative and glycation metabolism, providing a transcriptional booster to the biochemical actions of RSV and CeO_2_. This nanoparticle system demonstrated high biocompatibility and sustained bioactivity from the drug delivery point of view, accompanied by efficient intracellular uptake. Stability in biological media and a modular architecture also attest to the translational feasibility of this platform for future in vivo applications.

## 4. Discussion

### 4.1. Oxidative Stress in Cataractogenesis

Cataract formation is fundamentally based on oxidative damage. For example, the lens is constantly exposed to light and high oxygen concentrations in the aqueous humor, making it especially susceptible to oxidative stress. ROS can cause compositional changes in crystallin proteins to form insoluble aggregates, resulting in a loss of lens clarity. Antioxidants act by neutralizing this oxidative damage through scavenging ROS, thereby protecting the structural integrity of the lens. More recent data confirm this classical picture but add new layers of regulation. For instance, besides modulating ROS levels, NACA also modulates transcriptional activity for TXNIP and the inflammasome cascade, whereas β-casomorphin-7 and TMZ modulate Foxo1 and Nrf2, respectively, supporting the idea that redox modulation should be interpreted in the context of broader regulatory frameworks [15,16]. These agents illustrate the way endogenous antioxidant pathways can be pharmacologically modulated for therapeutic gain. The protective functions of antioxidants encompass the following: (1) direct neutralization of reactive oxygen species (ROS), (2) upregulation of endogenous enzymes (e.g., SOD, catalase), (3) epigenetic modulation (e.g., demethylation of Keap1), and (4) inhibition of protein glycation and aggregation. These mechanisms combine to stabilize lens proteins, diminish lipid peroxidation, and preserve clarity.

An important caveat, though, is that ROS have a dual role in cellular physiology. Circulating high concentrations of ROS is destructive, but low concentrations of ROS are a part of normal cellular signaling and for homeostatic regulation. This multifaceted role means that oxidative stress supplementation must be carefully calibrated to ensure that physiological processes are not disturbed. The protective versus potentially counterproductive effects of antioxidants are still debated and highlight the need for the right dosing regimens and targeted delivery systems.

### 4.2. Challenges Related to Bioavailability and Delivery

One consistent theme within the literature is the difficulty in allowing supplemented antioxidants to build in physiologically relevant concentrations that reach the lens. The lens is an avascular organ, obtaining nutrients from the aqueous and vitreous humors through diffusion and active transport. Braakhuis et al. explained the complexities brought into delivering exogenous antioxidants by its distinctive microcirculation in the lens [6]. A systemic boost in an antioxidant does not ipso facto mean a higher concentration in the lens nucleus, where oxidative damage is most potent.

New methods of delivery speak directly to this issue. Ishida et al. showed that topical NACA could preserve lens clarity in vivo, suggesting that local ocular delivery can bypass the limitations of systemic delivery [15]. Similarly, intravitreally administered LAN-NPs led to a long-lasting intraocular presence and structural restoration in early cataracts [13]. These formulations form a promising horizon for clinical translation.

### 4.3. Evidence from Laboratory Studies

Preclinical research continues to shape our understanding of cataract pathogenesis and the development of treatments. In vitro models of cultivated LECs and ex vivo and in vivo animal models have shown that exposure to oxidative stressors, such as hydrogen peroxide or UV light, causes crystallin oxidation, protein aggregation, and apoptosis. Treatment with different antioxidants dramatically attenuates these effects, underscoring the importance of redox balance in maintaining clear lenses. Recent work has identified further mechanisms, where specific molecular pathways have come to the forefront in antioxidant protection.

TMZ has been shown to protect against selenite-induced cataractogenesis both in vitro and in vivo by enhancing Nrf2 expression and preventing the demethylation of the Keap1 promoter, thereby preserving antioxidant defense [10]. The Keap1/Nrf2 axis, a master regulator of oxidative stress responses, might be confirmed as a targetable node with both transcriptional and epigenetic relevance.

MOSE has been shown to substantially prevent H_2_O_2_-induced lens opacity in cultured mouse lenses not solely through its radical-scavenging ability, but rather through its activation of endogenous antioxidant systems (GSH, SOD, and CAT) and upregulation of PPARα, a nuclear receptor involved in regulating the transcription of antioxidant genes [11]. While MOSE showed a less direct free radical-scavenging capacity than the control flavonoid luteolin in cell-free systems, it provided greater protection in the organ culture model; this suggested that its mechanism of action was based on more than direct antioxidant activity but also involved cellular redox regulation and transcriptional control.

The study suggested that the protective effects of MOSE might not be solely due to its radical-scavenging ability, but rather through its activation of endogenous antioxidant systems and upregulation of PPARα, which is involved in regulating the transcription of antioxidant genes. Their findings indicated that MOSE might serve as a promising, low-cost natural compound for preventing oxidative stress-induced cataractogenesis. Its effect on both biochemical antioxidants and transcriptional regulation via PPARα could make it particularly suitable for further development as a nutraceutical or complementary intervention for cataract prevention.

Significant methodological contributions to preclinical lens research by offering a repeatable methodology for assessing anti-cataract drugs using combined optical and biochemical endpoints, in addition to its therapeutic implications, have been reported in the literature [12]. Notably, they established and validated a new ex vivo model for cataract research using cultured bovine lenses, standardizing the use of light transmittance measurement as a quantitative marker for lens opacity. Using H_2_O_2_ to induce cataractogenesis, the study demonstrated a clear, dose-dependent decline in lens transparency accompanied by significant reductions in antioxidant enzyme levels, specifically GSH and SOD.

Treatment with L-cysteine significantly mitigated these changes by preserving lens transmittance and restoring GSH and SOD activity, thereby confirming its efficacy as a benchmark antioxidant in this model. The findings demonstrated that L-cysteine has better and longer-lasting protective benefits than ascorbic acid, especially in situations of chronic oxidative stress. Their results also indicated that lens spectral transmittance at 420 nm might serve as a quantitative surrogate for cataract severity, offering a reproducible and non-invasive alternative to conventional grading systems. The authors suggested that this method might be broadly applicable for screening anti-cataract compounds, particularly those targeting oxidative stress-related pathways. The approach might offer a cost-effective and scalable platform for preclinical pharmacological testing in cataract prevention research.

The intravitreal injection of lanosterol nanoparticles (LAN-NPs) has been shown to ameliorate, at least in part, the early structural collapse of the lens and delay cataract progression in rats [13]. They also prepared a nanoparticle formulation of lanosterol using bead milling, and their results showed that they achieved sustained delivery of lanosterol to the lens with no signs of toxicity or ocular irritation. In non-cataractous rats (SCR-N), LAN-NPs reversed early spatial and structural lens collapse, while in cataractous rats (SCR-C), repeated LAN-NP injections attenuated biochemical markers associated with lens opacification, including calcium accumulation, nitric oxide production, lipid peroxidation, and calpain activity.

Unlike prior work suggesting the reversal of cataract opacity in dogs, the current results suggested that LAN-NPs might only be effective at the pre-opacification stage, offering structural protection but not therapeutic reversal of established damage. Differences in lens composition, species, and delivery methods could explain these discrepancies. These findings suggested a structural stabilization strategy for cataract prevention, in which lanosterol delivery might mitigate downstream oxidative and proteolytic damage that contributes to cataractogenesis, particularly when administered before severe collapse has occurred. The authors proposed that future work could enhance lanosterol bioavailability and explore nanoparticle-based eyedrop formulations as a non-invasive alternative.

Ginsenoside Rg1 has been reported to significantly reduce H_2_O_2_-induced lens opacity in an ex vivo rat lens model as a result of strengthening endogenous antioxidant capacities [14]. The protective effects were attributed not only to free radical scavenging but also to the modulation of intracellular redox balance. Rg1 might have also functioned via hormone-like mechanisms, acting as a phytoestrogen, which has previously been associated with decreased cataract risk. Rg1 also enhanced cell viability in LECs subjected to oxidative stress, indicative of a potential cytoprotective role likely mediated by antioxidative and anti-apoptotic effects. The results provide novel insights that support Rg1 as a potential new pharmacological agent for cataract prevention related to localized reinforcement of the antioxidant system and protection of cellular function, especially through local (topical) administration, due to its water solubility, low toxicity, and multifaceted antioxidant activity.

NACA has been reported to exert a dual modulation of oxidative and inflammatory pathways, thus reducing ROS levels in H_2_O_2_-stressed LECs and downregulating TXNIP, thereby preventing NLRP3 inflammasome activation and downstream inflammatory responses [15]. NACA decreased H_2_O_2_-induced opacity in ex vivo lens cultures while preserving lens structure. NACA also mitigated lens opacity in aged mice when administered topically, and mortality and Txnip expression correlated with the promotion of redox homeostasis. NACA also demonstrated higher bioavailability and lipophilicity compared to classical antioxidants, translating into superior cellular availability and therapeutic effects, making it promising as a non-invasive, topical remedy for senile cataracts.

β-casomorphin-7 (β-CM-7), a milk-derived peptide, has been reported to increase resistance of HLECs to oxidative damage through modulation of important transcriptional regulators for an antioxidant defense [16]. β-CM-7 increased the expression and nuclear localization of Foxo1, an important transcription factor gene related to oxidative stress resistance and simultaneously caused upregulation in nuclear localization of SP1 genes, which could cooperate with Foxo1, which supports the possibility of the involvement of the Foxo1/Sp1 signaling axis in the antioxidant defense mechanism. The transcriptional changes were correlated with the upregulation of GSH-px, an antioxidant enzyme acting downstream.

Pretreatment with β-CM-7 enhanced the viability of cells, reduced intracellular levels of ROS and MDA, and restored activity of SOD. This study demonstrated that β-CM-7 might serve as a novel bioactive peptide with the potential to delay or prevent diabetic cataract progression. It enhanced endogenous antioxidant responses and moderated nuclear transcription factors associated with redox balance, making it a candidate for a potentially safe and natural therapeutic for diabetic ocular complications. The authors also proposed topical delivery or systemic supplementation for future studies.

Sustained HG exposure can lead to dynamic dysregulation of autophagy and oxidative stress in LECs, contributing to diabetic cataractogenesis [17]. Using both streptozotocin-induced diabetic mice and HG-treated mouse LECs in vitro, the study demonstrated that autophagy is a double-edged mechanism: it was initially activated in an adaptive manner), but later it became susceptible to inhibition under chronic stress, suggesting that sustained hyperglycemia might initially trigger a compensatory autophagy response, which could later become exhausted or inhibited. In parallel, oxidative stress markers SOD2 and CAT were transiently upregulated before a significant decline under prolonged HG conditions, reflecting an initial compensatory antioxidant response followed by redox failure. Importantly, pharmacologic induction of autophagy with rapamycin reduced mitochondrial ROS generation and restored antioxidant enzyme expression, whereas autophagy inhibition with chloroquine aggravated oxidative injury. These findings highlight that a therapeutic enhancement of autophagy, potentially via agents like rapamycin, might mitigate oxidative injury in LECs and delay progression.

A multifunctional nanotherapeutic strategy has been reported to prevent diabetic cataracts by engineering CeO_2_ NPs co-loaded with RSV and exosomal miRNAs [18]. This composite system of miRNA-RSV-CeO_2_ NPs exhibited enhanced redox activity, antiglycative efficacy, and gene-regulatory potential. In vitro, it outperformed unmodified CeO_2_ and RSV-CeO_2_ formulations in suppressing oxidative stress and protein crosslinking. The nanocarrier protected HLECs from oxidative apoptosis and upregulated CRYAA gene expression, a critical chaperone involved in maintaining lens clarity. The system’s therapeutic benefit was attributed to the synergistic action of RSV’s antioxidant/glycation-inhibiting properties, CeO_2_’s redox cycling (Ce^3+^/Ce^4+^), and the gene-modulatory capacity of exosomal miRNAs targeting oxidative and glycation pathways. The authors concluded that the miRNA-RSV-CeO_2_ NP system might be superior to the regular single antioxidant treatments and reveal new perspectives for the lens multifunctional protective strategies by simultaneously addressing the questions of ROS generation and protein glycation, as well as gene dysregulation.

Adding DC models, like the ones induced by streptozotocin, further expands on this ROC (antioxidant of choice) insight. These models mimic aspects of the metabolic stresses experienced in patients with diabetes and show that hyperglycemia exacerbates oxidative stress in the lens. Studying both senescent DNA damage and DCs may provide insight into a wider array of oxidative insults and the potential therapeutic benefit of antioxidants under various experimental settings. This strategy also mirrors the real-world clinical complexity, whereby aging and diabetes frequently overlap as comorbidities, which makes a singular-model perspective insufficient to capture the entire disease burden.

### 4.4. Evidence from Observational Studies

Several observational studies have reported strong associations between higher dietary intakes of antioxidants and decreased risk of cataract. Lutein and zeaxanthin, carotenoids found in the lens and macula, can absorb blue light and neutralize singlet oxygen species. These chemicals have demonstrated an inverse correlation with cataract risk in numerous epidemiological studies. Jiang et al. showed that in cohort studies, increasing consumption of vitamin A, vitamin C, vitamin E, β-carotene, and the carotenoids lutein and zeaxanthin was associated with a significant decrease in the risk of developing cataracts [5]. The reduction in the risk of cataracts was dose-dependent, with especially strong associations seen for vitamin C and lutein/zeaxanthin. Likewise, the inverse correlation between the consumption of antioxidant-rich fruits and vegetables and the occurrence of age-related cataracts has been reported by Sella et al. [2]. The scientists emphasized that accumulated exposure to dietary antioxidants over a lifetime seems to play a key role in keeping the lens clear. Moreover, higher dietary intake of antioxidant nutrients, such as zinc and the polyphenols, vitamins C and E, was linked to a significantly lower 10-year risk of developing nuclear cataracts in older adults, according to the Blue Mountains Eye Study, a large prospective cohort study carried out in Australia [19]. Fruits, vegetables, tea, coffee, and wine all contain naturally occurring polyphenols, which are well-known for their potent anti-inflammatory and antioxidant qualities. By scavenging ROS and modifying redox-sensitive cellular pathways, they reduce oxidative stress, which is linked to the development of lens opacity. As a cofactor for enzymes like SOD and by protecting proteins and cell membranes from oxidative damage, zinc supports antioxidant defense. Additionally, it works in concert with antioxidant vitamins to help transport and bioactivate vitamin A and to help regenerate oxidized forms of vitamins C and E [20]. Additional cross-sectional data from the National Health and Nutrition Examination Survey (NHANES) indicated that a higher Composite Dietary Antioxidant Index (CDAI), incorporating zinc, was inversely associated with cataract prevalence among U.S. adults aged 50 and older [21]. Together, these findings suggest that both zinc and polyphenols may play complementary roles in preserving lens transparency through nutritional modulation of oxidative stress. However, these studies have inherent limitations, such as reliance on self-reported dietary intake and confounding variables, which restrict the extent to which the protective effects can be attributed exclusively to the antioxidants under investigation, despite the promise indicated by observational data. Further longitudinal studies are needed to establish causality.

### 4.5. Evidence from Intervention Trials

Interventional studies, especially RCTs, offer a more strenuous test of the hypothesis that antioxidant supplementation may delay cataract progression. However, the evidence from these trials has been mixed. The consensus is that vitamin E or carotenoid supplementation has not consistently been shown to reduce the need for cataract surgery or to significantly alter the course of the disease, although modest effects have been reported in some RCTs. This lack of concordance between observational data and interventional outcomes may reflect, in part, the low bioavailability of orally administered antioxidants to reach the lens in drug-like concentrations. As also shown in recent preclinical studies, systemic delivery alone is likely insufficient, especially when the target tissue is avascular and shielded by physical barriers. Interventional trials have produced poor or variable outcomes, frequently because of the low absorption of systemic formulations. Innovative delivery methods such as topical NACA and intravitreal LAN-NPs may surmount these restrictions by facilitating lens-targeted activity.

The effectiveness of NACA as a topical agent and LANNPs as an intravitreal injection that acts as a delivery system are critical for therapeutic efficacy. They are particularly relevant given the disappointing outcomes from prior clinical trials using systemic delivery. Braakhuis et al. critically reviewed the methods of antioxidant delivery and metabolism in the lens [6]. The authors emphasized that the lens’s unique internal microcirculation system, responsible for the active transport of nutrients and antioxidants, is a key determinant of the dietary intervention’s bioefficacy. The review indicates that neglecting how this network functions in the gut may have played a role in the mixed outcomes of some clinical trials.

### 4.6. Additional Considerations: Broader Dietary Bioactives and Past Reviews

This review concentrates on nutrient-based antioxidant interventions for age-related and diabetic cataracts from the past five years; however, it is essential to recognize the substantial research conducted prior to this timeframe that underscores the therapeutic potential of a broader spectrum of dietary elements. Numerous thorough reviews released before 2018 have delivered detailed overviews of the significance of antioxidants and other nutrients in ocular health and cataract prevention, thereby contextualizing the more recent findings presented here [22,23].

A substantial body of research has focused on plant-derived chemicals and food supplements abundant in antioxidants, many of which are currently marketed commercially. Anthocyanins, a category of flavonoids prevalent in berries such as blueberries, bilberries, and blackcurrants, have attracted significant interest due to their powerful antioxidant and anti-inflammatory effects. Epidemiological and experimental research indicate that heightened intake of anthocyanin-rich foods or supplements may correlate with a diminished risk of cataract onset and progression [24,25]. The preventive functions of anthocyanins are believed to include the neutralization of reactive oxygen species, safeguarding lens epithelial cells from oxidative harm, and the modulation of intracellular signaling pathways [24].

In addition to anthocyanins, omega-3 polyunsaturated fatty acids (PUFAs), specifically eicosapentaenoic acid (EPA) and docosahexaenoic acid (DHA) present in fish oil, have been examined for their possible contribution to cataract prevention. Although the precise mechanisms remain under investigation, omega-3 polyunsaturated fatty acids are essential constituents of cell membranes and are recognized for their anti-inflammatory and neuroprotective properties. Certain studies indicate that increased dietary consumption or supplementation of omega-3 PUFAs may correlate with a reduced occurrence of age-related cataracts, maybe by alleviating oxidative stress and inflammation in the lens [26,27]. Nonetheless, similar to most nutritional therapies, findings from several studies have been inconsistent, underscoring the necessity for additional large-scale, well controlled clinical trials.

Moreover, the functions of recognized antioxidants like lutein and zeaxanthin, together with vitamins C and E, have been thoroughly documented in prior studies, establishing the fundamental comprehension of how dietary antioxidants may enhance lens health [5,20,22]. The nutrients, concentrated in the lens, are essential for safeguarding against photo-oxidative damage and preserving lens transparency. Numerous commercially available ocular health supplements are designed based on studies supporting these particular micronutrients.

The investigation of several plant extracts persists in producing potential options for cataract prevention. Compounds derived from several botanical sources have exhibited antioxidant, anti-glycation, and aldose reductase inhibitory effects in preclinical models [28]. The current analysis concentrated on particular, newly studied compounds; nonetheless, the extensive realm of phytotherapy for cataracts necessitates continued research to discover and substantiate new treatment candidates.

## 5. Conclusions

Currently, the available research suggests a potentially beneficial but also dose-responsive relationship between antioxidant intake and cataract prevention.

Oxidative damage may selectively impact various parts of the lens. Nuclear cataracts have been mostly related to persistent oxidative stress and aging, whereas cortical cataracts have been frequently linked to UV exposure. The evaluated agents, particularly lanosterol and cerium oxide nanoparticles, seemed to exhibit greater efficacy in early-stage cortical and nuclear cataracts. Nevertheless, there is insufficient information about the specific efficiency of antioxidants according to the type of cataract, especially in posterior subcapsular cataracts, highlighting the necessity for focused investigation. Observational studies consistently suggest a protective role of dietary antioxidants, but RCTs produced mixed results, potentially due to methodological issues and problems of bioavailability. More fine-tuned strategies that can provide concurrent targeted delivery, transcriptional modulation, and structural stabilization should be developed to realize the full therapeutic potential of antioxidant interference. Despite the important finding that antioxidants were themselves genetic determinants of lens health, we have yet to overcome major barriers to translating these findings into successful clinical therapeutics. Recent studies have broadened our view of the antioxidant actions at a molecular scale, illustrating how the antioxidant response in n cells is expanded beyond ROS and together involves transcriptional regulators as well as inflammatory pathways.

New technologies with improved delivery capacity, such as topical application or nanoparticle associated delivery, are beginning to directly address anatomical challenges that have restricted the efficacy of systemically distributed interventions [11,12]. Our review emphasizes the importance of a holistic strategy in this area of research and can serve as a basis for further studies aimed at alleviating the worldwide burden of age-related and diabetic cataract.

## Data Availability

Data are contained within the article.

## Abbreviations

The following abbreviations are used in this manuscript:

DC	diabetic cataract
AGEs	advanced glycation end products
UV	ultraviolet
ROS	reactive oxygen species
RCTs	randomized controlled trials
TMZ	trimetazidine
HLEB3	human lens epithelial B3
SOD	superoxide dismutase
GPx	glutathione peroxidase
CAT	catalase
MDA	malondialdehyde
NO	nitric oxide
H_2_O_2_	hydrogen peroxide
MOSE	Moringa oleifera stem extract
GSH	glutathione
DPPH	2,2-Diphenyl-1-1-picrylhydrazyl
DMEM	Dulbecco’s Modified Eagle’s medium
AA	ascorbic acid
LAN-NPs	lanosterol nanoparticles
SCRs	Shumiya Cataract Rats
HLEC	human lens epithelial cells
LPO	lipid peroxidation
LECs	lens epithelial cells
WSP	water-soluble protein
NAC	*N*-acetylcysteine
NACA	*N*-acetylcysteine amide
MLECs	mouse lens epithelial cells
TXNIP	thioredoxin-interacting protein
β-CM-7	β-casomorphin-7
MTT	4, 5-dimethyl-2-thiazolyl)-2, 5-diphenyl-2-H-tetrazolimol/L bromide
CeO_2_ NPs	cerium oxide nanoparticles
RSV	resveratrol
miRNAs	microRNAs

## Appendix A

**Table A1:** The table summarizes the included articles.

Agent	Study Model(s)	Key Outcomes	Mechanism of Action	Relative Efficacy	GRADE ^1^	OCEM Level ^2^
Trimetazidine (TMZ)	In vivo (rat), In vitro (HLECs)	Reduced lens opacity, decreased ROS, preserved Bcl-2/Bax ratio	Epigenetic modulation (Keap1 demethylation), Nrf2 pathway activation	High	Low	4
*Moringa oleifera*	Ex vivo (mouse lens)	Maintained lens transparency, improved antioxidant enzyme levels	Upregulation of PPARα, increased GSH, SOD, and CAT	Moderate–High	Low	4
L-Cysteine	Ex vivo (bovine lens)	Preserved light transmittance, restored GSH and SOD activity	Restored sulfhydryl pools, stabilized antioxidant enzymes	Moderate	Low	4
Lanosterol NPs	In vivo (Shumiya Cataract Rats)	Reversed early lens collapse, reduced biochemical markers of cataract	Prevented crystallin aggregation, reduced LPO and calcium accumulation	Moderate	Low	4
Ginsenoside Rg1	Ex vivo (rat lens), In vitro (HLECs)	Reduced lipid peroxidation, increased soluble proteins, protected lens cells	Enhanced antioxidant enzyme activity, reduced MDA, increased GSH and SOD	High	Low	4
NACA/NAC	In vitro, ex vivo, in vivo (aged mice)	Preserved lens clarity, reduced TXNIP expression, improved redox balance	Suppressed inflammasome activation, restored thioredoxin system	Very High	Low	4
β-Casomorphin-7	In vitro (HLECs under hyperglycemia)	Reduced ROS and MDA, restored FoxO1 and Sp1 expression	Activation of nuclear transcription factors, enhancement of antioxidant enzymes	Moderate–High	Very Low	5
Rapamycin	In vivo (diabetic mice), in vitro (mouse lens epithelial cells)	ROS reduction and preserved mitochondrial integrity	Autophagy modulation by rapamycin	Moderate–High	Low	4

HLEC = human lens epithelial cells; ROS = Reactive Oxygen Species; MDA = Malondialdehyde; AGEs = Advanced Glycation End-products; RSV = Resveratrol; CeO_2_ = Cerium Oxide; miRNA = microRNA; CRYAA = α-Crystallin A; MLEC = mouse lens epithelial cells; RT-qPCR = Real-Time Reverse Transcriptase-Quantitative Polymerase Chain Reaction; NACA = N-Acetylcysteine Amide; TXNIP = Thioredoxin-Interacting Protein; TRX = Thioredoxin; WSP = Water-Soluble Protein; Rg1 = Ginsenoside Rg1; GSH = Glutathione; SOD = Superoxide Dismutase; MDA = Malondialdehyde; H_2_O_2_ = Hydrogen Peroxide; HLEB3: human lens epithelial B3; WB = Western blot; TMZ = Trimetazidine; Nrf2 = Nuclear factor erythroid 2–related factor 2; Keap1 = Kelch-like ECH-associated protein 1; CAT = Catalase; GPx = Glutathione Peroxidase; TEM = Transmission Electron Microscopy; MOSE = Moringa oleifera Stem Extract; PPARα = Peroxisome Proliferator-Activated Receptor Alpha; MTT = 5-diphenyl-2-H-tetrazolimol/L bromide; β-CM-7 = Beta-Casomorphin-7; GSH-px = Glutathione Peroxidase; Foxo1 = Forkhead box protein O1; Sp1 = Specificity Protein 1; SCR-N = Shumiya Cataract Rat non-cataractous rat; SCR-C: Shumiya Cataract Rat cataractous rat; LPO = Lipid Peroxidation; LAN-NPs = Lanosterol Nanoparticles. ^1^ Grading of Recommendations Assessment, Development and Evaluation (GRADE), ^2^ Oxford Centre for Evidence-Based Medicine (OCEM) 2011 guidelines. The relative efficacy of each antioxidant agent has been defined as Very High: Multifaceted mechanism, robust outcomes across multiple models; High: Consistent improvement in lens transparency and redox homeostasis; Moderate–High: Demonstrated efficacy in key parameters but limited in scope or model; Moderate: Effective in early-stage cataract or partial restoration.
